# Measles outbreak: preliminary report on a case series of the first 8,070 suspected cases, Manaus, Amazonas state, Brazil, February to November 2018

**DOI:** 10.2807/1560-7917.ES.2019.24.2.1800663

**Published:** 2019-01-10

**Authors:** Guilherme Almeida Elidio, Giovanny Vinícius Araújo de França, Flávia Caselli Pacheco, Marinélia Martins Ferreira, Jair dos Santos Pinheiro, Eliane Nogueira Campos, Bernardino Cláudio de Albuquerque, Rosemary Costa Pinto, Angela Desiree Carepa Santos da Silva, Priscila Leal e Leite, Greice Madeleine Ikeda do Carmo, Andre Luiz de Abreu, Cintia Paula Vieira Carrero, Marli Rocha de Abreu, Fabiano Marques Rosa, Cesar M. de Oliveira, Dirce Bellezi Guilhem

**Affiliations:** 1 Secretariat of Health Surveillance, Ministry of Health, Brasília, Brazil; 2 Municipal Health Secretariat of Manaus, Amazonas, Brazil; 3 Fundação de Vigilância em Saúde do Estado do Amazonas, Manaus, Brazil; 4 Department of Epidemiology & Public Health, University College London, London, UK; 5 Post-Graduation Program in Health Sciences, University of Brasília, Brasília, Brazil

**Keywords:** measles, communicable diseases, epidemiology, disease outbreaks, America, Brazil, airborne infections, viral infections, outbreaks

## Abstract

We report an ongoing measles outbreak in Manaus, Amazonas state, Brazil. As at 3 November 2018, 1,631 cases were confirmed corresponding to an incidence of 75.3 per 100,000 inhabitants; all five sanitary districts presented confirmed cases. Reintroduction of measles virus in Manaus is likely related to the current outbreak in Venezuela and due to recent decline in measles vaccine coverage. Given the current scenario, prevention and control measures should target individuals aged 15–29 years.

In February 2018, a measles outbreak was reported in the state of Roraima, Brazil and was found to be associated with the ongoing measles outbreak in Venezuela [1]. As at 10 December 2018, 349 cases have been confirmed [2]. Between 2001 and 2017, the Amazonas state did not report any confirmed measles cases [3]. Here, we report the ongoing measles outbreak in Manaus capital of the Amazonas state in Brazil. The north of Amazonas state borders Venezuela. Manaus has 2,145,444 inhabitants.

## Case definition, laboratory testing and investigations

In 1969, the Ministry of Health (MoH) in Brazil established that all suspected measles cases seeking health services must be notified to the local authorities; cases must also be registered in the National System of Notifiable Diseases (Sinan) [4]. In addition, an ad hoc system known as ‘TRACK’ was made available in Manaus during the epidemiological week (EW) 19 (starting on 12 May 2018) to track register all data collected in the health services and through interviews in the present outbreak. In this study, we included all cases registered in TRACK until 3 November 2018 who lived in Manaus and met the suspected case definition (Box) [5-7].

Box **Definition of cases and other categories followed-up during the measles outbreak, Manaus, Amazonas, Brazil, 2018**

**Suspected case:**

• Any person in whom a health professional suspects measles infection; or

• Any person with fever and maculopapular rash (i.e. non-vesicular) and cough, coryza (i.e. runny nose) or conjunctivitis (i.e. red eyes); or

• Any person with a history of travel to sites with measles virus circulation in the last 30 days, or who has had contact in the past 30 days with someone who has travelled to sites with viral circulation.

**Confirmed case:**

Laboratory: A case that meets the clinical case definition and has laboratory-confirmation of measles virus infection:

• A positive serologic test for measles immunoglobulin M antibody; or

• Isolation of measles virus from a clinical specimen; or

• Detection of measles-virus specific nucleic acid from a clinical specimen using polymerase chain reaction; or

• IgG seroconversion or a significant rise in measles immunoglobulin G antibody using any evaluated and validated method.

Epidemiologically: A case that meets the clinical case definition and has contact with a laboratory-confirmed measles case whose rash onset was within the preceding 21 days, or a case that lives in the same district or adjacent districts where a measles outbreak has been laboratory- confirmed and transmission is plausible.

**Discarded case:**

A suspected case, which, upon adequate investigation that includes a blood specimen collected in the appropriate time frame, lacks serologic evidence of a measles virus infection.

In Brazil, all samples processed by the public health laboratory network are registered in the Laboratory Environment Management System (GAL); test results obtained in the ongoing outbreak available in GAL were also registered in TRACK to support case classification. The epidemiological and laboratory investigations, as well as contact tracing, were conducted by the Secretariat of Health of the municipality of Manaus, together with the Amazonas State Health Secretariat and the Brazilian MoH. All samples were sent to the National Reference Laboratory at the Oswaldo Cruz Foundation (Fiocruz) in Rio de Janeiro. Those who met the clinical criteria and presented laboratory confirmation were considered as ‘confirmed’.

## Outbreak onset

The first laboratory confirmed case of the ongoing outbreak was a woman in her early 20s living in the northern district of Manaus. She had rash onset on 21 February 2018 and presented with fever, cough, coryza and conjunctivitis. She was identified during the investigation of her one-year old child’s case who had rash onset on 1 March and presented the same symptoms as the mother. Laboratory confirmation of measles was obtained on 23 March 2018.

The D8 genotype measles virus was identified in a urine sample from the infant, which was identical to the one that is circulating in Venezuela; genotyping also showed that it was a wild-type measles virus. The mother reported having had contact with three Venezuelan migrants in January 2018, whom were interviewed by the surveillance team; one of them presented with malaise, diarrhoea, high fever and redness in the body soon after arriving in Manaus, but denied having presented with measles rash. None of the migrants had a history of vaccination against measles. The migrants did not match the case definition for notification and were identified retrospectively, 40 days after their symptom onset, therefore, laboratory tests were not carried out as too much time had elapsed. The infant did not have contact with the migrants during the incubation period; the only contacts were the mother and father, and the latter had been vaccinated against measles. Therefore, the probable source of infection of the infant was the mother.

Although the current available information suggest a link between the outbreaks in Venezuela and Manaus, further molecular epidemiological studies are needed to help understanding the reintroduction of measles in the municipality.

## Epidemiological situation

From 6 February–3 November 2018, 8,070 suspected cases were notified; of these, 5,971 (74.0%) were still under investigation. Among the cases with complete investigation, 1,631 (77.7%) were confirmed and 468 (22.3%) were discarded; the median interval between the date of notification and the date of the final classification of the case was 37 days (range 0–140 days). All the confirmed cases were Brazilians. The incidence of measles among confirmed cases was 75.3 per 100,000 inhabitants; the incidence was higher among infants (1,003.9/100,000 inhabitants) and children aged 1–4 years (175.3/100,000 inhabitants). Up to 3 November, the northern district accounted for the largest number of cases (2,753 cases) (Table 1).

**Table 1:** Key features of reported and confirmed measles cases, Manaus, Amazonas state, Brazil, 6 February–3 November 2018

Variable	Notified cases^a^ N = 7,602			Confirmed cases N = 1,631		
	n	%	Incidence per 100,000 inhabitants	n	%	Incidence per 100,000 inhabitants
**Sanitary district**						
East	2,454	32.3	460.1	525	32.2	98.4
North	2,753	36.2	461.5	506	31.0	84.8
West	1,011	13.3	211.3	290	17.8	60.6
South	1,325	17.4	253.4	270	16.6	51.6
Rural	59	0.8	418.2	40	2.5	283.5
**Sex**						
Female	3,385	44.5	355.2	755	46.3	79.2
Male	4,217	55.5	463.9	876	53.7	96.4
**Age group**						
< 6 months	549	7.2	2828.6^b^	203	12.5	1003.9^b^
6–11.9 months	578	7.6	2828.6^b^	197	12.1	1003.9^b^
1–4 years	746	9.8	489.9	267	16.4	175.3
5–9 years	314	4.1	160.1	100	6.1	51.0
10–14 years	355	4.7	166.3	116	7.1	54.4
15–19 years	1,770	23.3	853.1	225	13.8	108.4
20–29 years	2,021	26.6	467.3	332	20.4	76.8
30–39 years	830	10.9	229.0	101	6.2	27.9
40–49 years	319	4.2	113.8	61	3.7	21.8
50 + years	120	1.6	42.8	29	1.8	10.3
**Pregnancy**						
No	3,299	97.5	NA	744	98.5	NA
Yes	86	2.5	NA	11	1.5	NA
**Admitted to hospital**						
No	6,289	88.4	NA	1,164	71.9	NA
Yes	823	11.6	NA	455	28.1	NA
**Vaccination status**						
No	5,411	75.0	NA	1,288	82.9	NA
Yes	1,801	25.0	NA	265	17.1	NA
**Signs and symptoms**						
**Cough**						
No	494	6.5	NA	48	2.9	NA
Yes	7,101	93.5	NA	1582	97.1	NA
**Coryza**						
No	1,773	23.4	NA	235	14.4	NA
Yes	5,809	76.6	NA	1,393	85.6	NA
**Conjunctivitis**						
No	3,188	42	NA	509	31.4	NA
Yes	4,385	58	NA	1,114	68.6	NA

NA: not applicable.

^a^ Includes confirmed cases and those under investigation.

^b^ Incidence based on all infants < 1 year.

Source: Municipal Health Secretariat of Manaus, Amazonas, Brazil. Data extracted on 6 November 2018.

**Figure 1:**
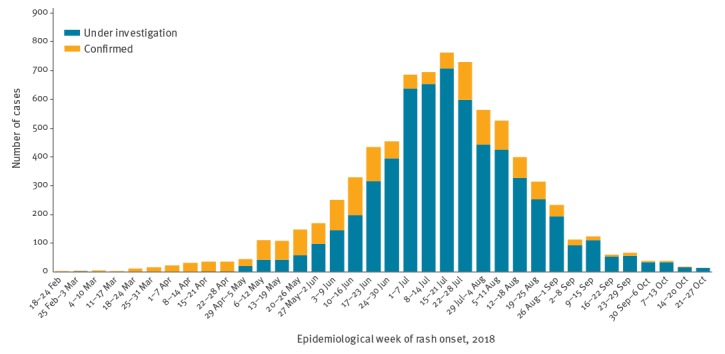
Distribution of reported cases of measles according to the epidemiological week of onset of symptoms and investigation status, Manaus, Amazonas state, Brazil, 6 February–3 November 2018

Source: Municipal Health Secretariat of Manaus, Amazonas, Brazil. Data extracted on 6 November 2018.

**Figure 2:**
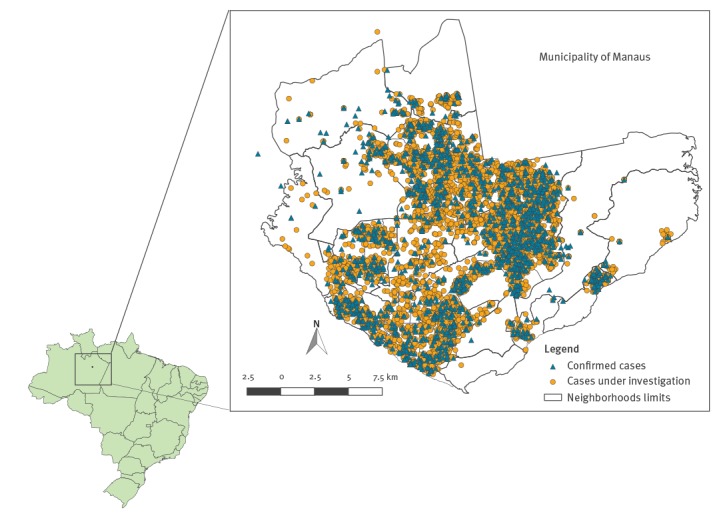
Spatial distribution of reported cases of measles according to investigation status, Manaus, Amazonas state, Brazil, 6 February–3 November 2018

Source: Municipal Health Secretariat of Manaus, Amazonas, Brazil. Data extracted on 6 November 2018.

The epidemic curve shows a gradual increase in the number of cases with symptom onset from 6 May, reaching a peak of 763 cases in EW 29 (15–21 July), then decreasing gradually (Figure 1). The median reporting delay was 1 day (range 0–155 days), considering the difference between the notification and rash onset dates; therefore, the decrease in the number of notifications from the EW 30 onwards does not seem to be explained by the reporting delay. All five districts presented confirmed cases, which indicates the circulation of the virus throughout the municipality (Figure 2).

**Figure 3:**
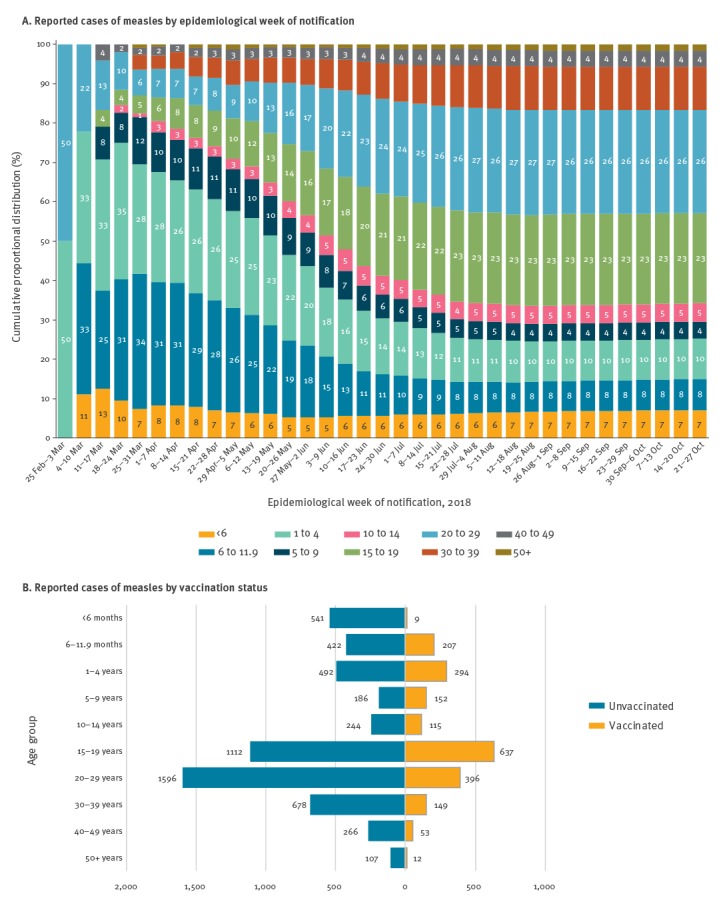
Proportional distribution of reported cases of measles according to age group, stratified by (A) epidemiological week of notification and (B) vaccination status^a^, Manaus, Amazonas, Brazil, 6 February–3 November 2018

MMR: measles, mumps, rubella; MMRV: measles, mumps, rubella, varicella.

^a^ Vaccination status is based on the calendar for national routine immunisation established by the Brazilian Ministry of Health, as follows: one dose of MMR vaccine administered at 12 months of age; one dose of tetravalent vaccine against MMRV at 15 months; two doses of MMR between 2–29 years of age; and one dose of MMR from 30–49 years of age.

Source: Municipal Health Secretariat of Manaus, Amazonas, Brazil. Data extracted on 6 November 2018.

Of 7,602 notified cases, the majority (55.5%) were male; among women, 86 of 3,385 (2.5%) were pregnant. The age group with the highest number of cases (26.6%) was the 20–29-year-olds, followed by those aged 15–19 years (23.3%). Together, these two age groups comprised ca 50% of all notifications (Table 1). Up to week EW 22, most cases were in the age group of 6 months–4 years, from the EW 23 onwards, however, an increase of cases aged 15–29 years was observed (Figure 3A).

Of 7,212 suspected cases, 75.0% had no records of vaccination against measles; the percentage was even higher among confirmed cases (82.9%) (Table 1). In addition, the majority of unvaccinated cases were in the age group of 15–29 years (Figure 3B).

Of the 7,602 suspected cases, 823 (11.6%) were hospitalised, and the average of the hospitalisation period was 4 days (Table 1). As of EW 29, two measles-related deaths had been recorded in the municipality of Manaus, both in male infants, aged 1 year or younger, below the age recommended for the first dose of measles vaccine, with no comorbidity; the D8 genotype was identified in one of the children and the other one is still awaiting genotyping.

## Brazil vaccination schedule

MCV has been available free of charge in Brazil since 1967; currently, routine vaccination follows the calendar established by the MoH: one dose of measles, mumps, rubella (MMR) vaccine administered at 12 months of age; one dose of tetravalent vaccine against measles, mumps, rubella, varicella (MMRV) at 15 months; two doses of MMR between 2–29 years of age; and one dose of MMR from 30–49 years of age [4,8].

Official data from the MoH indicate that the coverage of the first dose of MMR vaccine at 12 months of age decreased markedly in Manaus between 2014 and 2017 (105.7% vs 81.0%); the same pattern was observed for the second dose of the vaccine at 15 months of age in the same period (95.8% vs 67.0%) [9]. It is noteworthy that vaccination coverage in Brazil is obtained through an administrative method [10]; a coverage above 100% indicates that the number of doses administered in the municipality is greater than the number of residents in a specific age group and time period.

## Control measures

The management of the outbreak have been overseen by the MoH and coordinated by the local authorities. A situation room was established in Manaus in March 2018, in order to facilitate the coordination of all strategies for outbreak control and prevention. Since its creation, at least two deliberative meetings have been held weekly.

To date, several strategies have been adopted to interrupt the outbreak. Suspected cases have been investigated within 48 hours, searching for the source of infection and possible secondary cases. Extensive contact tracing for all measles cases has been conducted, verifying the immunisation status of contacts and vaccinating susceptible contacts within 72 hours. Self-isolation post exposure was also recommended. The epidemiological surveillance has been intensified through active and retrospective institutional case finding, including the identification of chains of transmission in the municipality. Initially, the local authorities requested immediate isolation of any suspected cases in reference hospitals. As the number of suspected cases increased substantially over time, hospitalisation became restricted to patients with complications given the limited availability of hospital beds.

Routine vaccination has been intensified in Manaus, free of charge for individuals aged 12 months–49 years. The vaccination campaign was anticipated, targeting children aged 12 months–5 years. It was conducted from 14–27 April 2018, and reached a coverage of more than 95% according to preliminary data provided by the MoH [11]. A national campaign was carried out from 6 August–31 October 2018, targeting individuals aged 12 months–5 years. Health professionals were hired and trained in case management and those with no record of vaccination against measles were vaccinated.

The laboratory network was strengthened, ensuring that samples were received at the reference laboratory within 5 days of collection. A risk communication strategy was implemented and media messages were disseminated, aiming to encourage vaccination when appropriate and advising the general population of the symptoms suggestive of measles. Epidemiological reports were made available weekly at the MoH webpage [12], as well as technical notes with guidelines on flows for epidemiological surveillance, laboratory and immunisation.

## Discussion

The concentration of cases in children aged 5 years and under at the beginning of the outbreak seems to be related to a recent decrease in the coverage of measles-containing vaccines in Manaus, which may have led to a marked increase in the number of individuals susceptible to the disease in this age group. Consequently, this favoured the reintroduction of the virus in the municipality. In this context, the national vaccination campaign targeting children aged 6 months–5 years seems to have been effective in controlling the spread of the virus in this age group. There was a change in the age distribution of the cases as the outbreak continued where more individuals aged 15–29 years were affected and the available data indicates that most of these individuals had no history of measles vaccination. As our findings are based on preliminary data, these observations should be further investigated in the future.

We acknowledge some limitations of our study. First, due to the magnitude of the outbreak it was not possible to carry out laboratory tests for all cases and most of the cases are still under investigation. Due to this, we opted to present the distributions of both notified and confirmed cases. In addition, there is need to consider the epidemiological link for case classification in future studies [13].

 Second, it was not possible to collect detailed vaccination histories to analyse, as surveillance teams only recorded whether an individual had been vaccinated with MMR and if so, the date of the last dose.

Our findings highlight the need to increase the awareness among health professionals so that they can better recognise and report suspected cases of measles. In addition, epidemiological surveillance for timely investigation of cases could be strengthened, as well as better identification of likely sources of infection and secondary cases. Furthermore, given the potential number of susceptible individuals in all age groups, it is essential to intensify routine vaccination efforts and to implement catch-up vaccination, especially for those individuals aged 15–29 years. At national level, the risk of spread in Brazil is high, taking into account the transmissibility of the disease and the performance of the routine immunisation programme. At the regional level, the potential impact is also considered high given the prevention and control capacities in other countries in the Region of the Americas [14,15]. Efforts made in recent years to eliminate measles in Brazil may be compromised if effective measures are not taken to stop the transmission of the virus in the northern region of the country.
